# Tailor-Made Bio-Based Non-Isocyanate Polyurethanes (NIPUs)

**DOI:** 10.3390/polym15061589

**Published:** 2023-03-22

**Authors:** Juan Catalá, Irene Guerra, Jesús Manuel García-Vargas, María Jesús Ramos, María Teresa García, Juan Francisco Rodríguez

**Affiliations:** Department of Chemical Engineering, Institute of Chemical and Environmental Technology, University of Castilla-La Mancha, Avda. Camilo José Cela 12, 13071 Ciudad Real, Spain

**Keywords:** NIPU, tailor-made, crosslink, cyclic carbonate, CSBO, priamine, vegetable oil

## Abstract

Non-isocyanate polyurethanes (NIPUs) based on biobased polyamines and polycarbonates are a sustainable alternative to conventional polyurethanes (PU). This article discloses a novel method to control the crosslinking density of fully biobased isocyanate-free polyurethanes, synthesized from triglycerides carbonated previously in scCO_2_ and different diamines, such as ethylenediamine (EDA), hexamethylenediamine (HMDA) and Priamine^TM^-1075 (derived from a dimerized fatty acid). As capping substances, water or bioalcohols are used in such a way that the crosslinking density can be adjusted to suit the requirements of the intended application. An optimization of the NIPU synthesis procedure is firstly carried out, establishing the polymerization kinetics and proposing optimal conditions set for the synthesis of the NIPUs. Then, the influence of the partial blocking of the active polymerization sites of the carbonated soybean oil (CSBO), using monofunctional amines, on the physical properties of the NIPUS is explored. Finally, the synthesis of fully biobased NIPUs with a targeted crosslinking density is achieved using hybrid NIPUs, employing partially carbonated oil and H_2_O or ethanol as blockers to achieve the desired physical properties in a very precise manner.

## 1. Introduction

The importance of the global polyurethane market is of remarkable scale. In 2020, its size exceeded USD 70.67 billion and is expected to reach USD 94.59 billion by 2029 [1]. This is mainly due to the wide range of utilities, such as foams, elastomers, adhesives, sealants and coatings, which enable their applications in diverse industries, including the biomedical, automotive and construction industries [2,3,4,5,6,7,8,9,10,11].

However, they also have drawbacks and the vast majority of them are associated with toxicity and environmental concerns. The main concern is related to the use of isocyanates, one of the monomers used in the synthesis of polyurethanes and industrially derived from phosgene, a highly poisonous gas at room temperature. Furthermore, within the hazards of the use of isocyanates, according to Annex XVII of the Registration, Evaluation, Authorization and Restriction of Chemicals (REACH), these are recognized as carcinogenic, mutagenic or toxic [12].

Finally, the processing of polyurethanes at the end of their life cycle can lead to combustion or landfill storage, where isocyanates are released and can decompose mainly to HCN in the case of combustion, or due to hydrolysis of the urethane bonds, toxic amines can be released in the case of landfilling [7]. In addition, the monomers (polyols, isocyanates and chain extenders) that constitute commercial polyurethanes are obtained from fossil resources with consequent environmental implications [13,14].

Considering all these drawbacks, alternatives such as non-isocyanate polyurethanes (NIPUs) are emerging. Research on innovative processes to manufacture NIPUs is becoming increasingly important due to the high industrial interest on these materials, due to the evident depletion of fossil feedstocks, the more exigent labor safety rules and the challenges associated with the inevitable advance of environmental concerns [15].

In addition, NIPUs may offer several advantages over conventional PUs due to the presence of several primary and secondary pendant hydroxyl groups in their backbone, as for the case of polyhidroxyurethanes, increasing the proportion of hydrogen bonds formed between the polymer chains themselves, creating the possibility of potential chemical modifications that give the products new properties and applications [16]. This can be reflected in the properties of these NIPUs, exhibiting increased water absorption capacity, thermal stability and chemical resistance [17,18]. Intensive non-isocyanate polyurethane research has also led to the development of hybrid NIPUs, in which NIPUs are obtained from modified precursors, such as partial carbonated epoxides or the addition of chain extenders and prepolymers, creating interpenetrated network structures based on polyhydroxyurethanes crosslinked with polyepoxides, with the ability to obtain materials with a sequence of soft and hard segments, such as in conventional polyurethanes [19,20].

**Figure 1:**
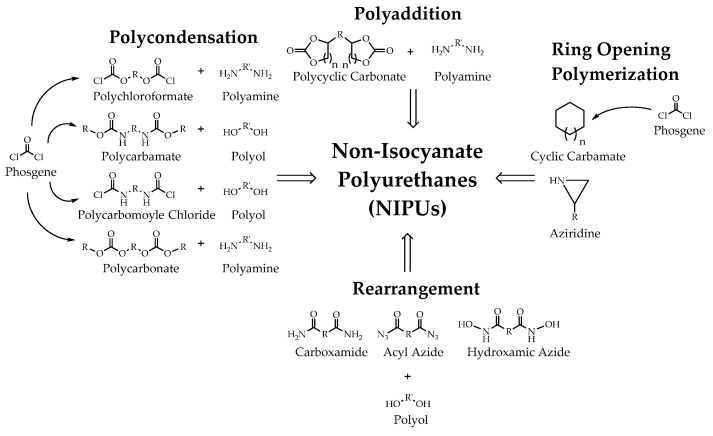
Main synthesis routes for NIPU synthesis.

The synthesis of this type of polyurethane represents a route towards sustainable chemistry. There are four main routes to obtain an NIPU: polycondensation, rearrangement, ring-opening-polymerization and polyaddition (Figure 1) [15].

The first three routes present a series of disadvantages of a rather similar nature. These problems are closely related to the toxicity involved in the need to use different phosgene derivatives, acylazides or carboxamides (making the elimination of isocyanate from the production process insufficient or irrelevant), to the generation of undesired by-products, such as alcohols or HCl, and, finally, to the requirement of working under extreme temperature conditions [15].

Hence, the preferred route would be the polyaddition of diamines and five-membered cyclic carbonates, which includes the advantage of not only eliminating isocyanates from the production process, but also the usage of different types of natural and renewable resources, such as vegetable oils and fats, and resources from starch, sugar or even wood [17,21,22,23,24,25,26,27].

In this sense, natural vegetable oils have shown to be a promising alternative as green precursors for NIPU synthesis, due to their convenient distribution of double bonds and ester bonds, ease of functionalization, large availability, affordable costs, and even in some casing being by-products [23,27,28]. There are also multiple new-generation green methods for their transformation, based on the epoxidation of the unsaturation naturally present in triglycerides [29,30], and subsequent carbonation with processes that also allow the capture and fixation of CO_2_ to their molecular structure, which was first discovered by Inoue et al. in 1969 [31], that has further demonstrated the evident advantages of making carbonating triglycerides [32,33,34,35].

Therefore, both the synthesis method and the nature of the reagents significantly affect the physical, chemical, mechanical, and structural properties of the NIPU, and these are defined by the application end-use. Thus, for instance, the length of the monomeric segments forming the chains or their nature or the crosslinking degree of a polyurethane is directly linked to its elasticity/rigidity, its foaming capacity or its thermal behavior, which divides polyurethanes into thermoplastics (TPU) and thermosets, depending on these factors [36,37,38,39].

**Scheme 1:**
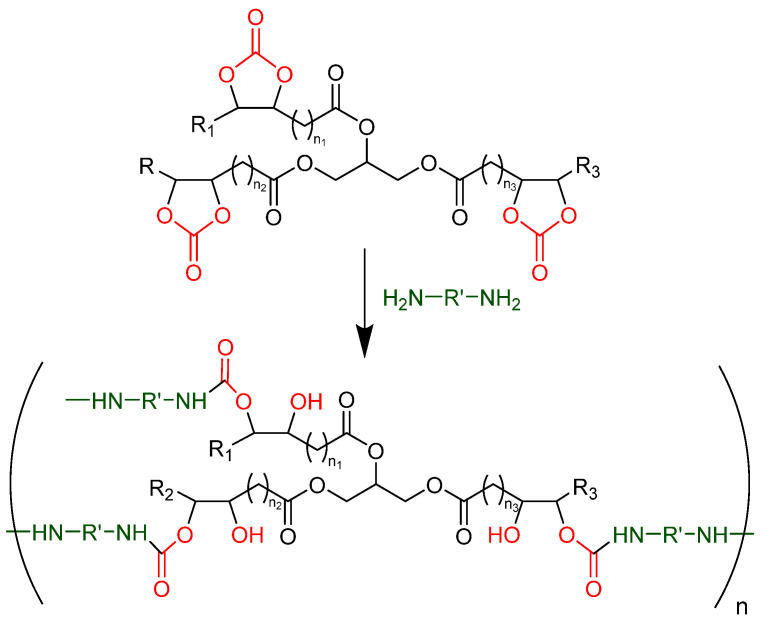
Polyaddition of carbonated triglycerides and diamines. Cyclic carbonate groups and derivatives are in red. Amine groups in green.

For that reason, the aim of this research is to delve deeper into the synthesis of different non-isocyanate polyurethane materials, in a way that allows control of the functionality, to obtain elastic materials, seeking properties similar to those of marketed products. As such, different-sized diamines (60–556 g·mol^−1^) have been used, as well as cyclic carbonates from the direct fixation of CO_2_ into the epoxidized product of soybean oil [40,41], which is represented in the general reaction scheme of a carbonated product of triglyceride nature (80–98% of the composition of vegetable oils) and a diamine (Scheme 1).

Thus, the goal of this paper is to produce renewable isocyanate-free polyurethanes by using carbonated triglycerides that have previously been produced in scCO_2_ with a crosslinking density that may be tailored to the most practical or desirable application.

Given this, the primary contribution of this study is the modification of the chemical structure of the initial resources, which affects their functionality (f) or quantity of available cyclic carbonate moieties, both of which have an effect on the crosslinking degree of the resulting polyurethane polymeric chains. Notwithstanding that the topic of non-isocyanate polyurethanes has been already explored, the main novelty of our work comes from the now demonstrated possibility to produce entirely biobased NIPUs with complete crosslinking control, allowing for the production of PUs with specific properties and even the obtainment of NIPU foams.

## 2. Materials and Methods

### 2.1. Materials

Carbonated soybean oil (CSBO), (f_average_ = 4.37, MW_average_ = 1190 g·mol^−1^ was synthesized from epoxidized soybean oil (ESBO), Traquisa S.L. (Barcelona, Spain), with an average molecular weight of 998 g·mol^−1^, following the methodology described in previous publications [41].

Priamine^TM^ 1075 dimer diamine (f = 2, dimer content: ≥99%; 556.5 g·mol^−1^) was kindly provided by Croda Ibérica S.A. (Barcelona, Spain).

Ethylenediamine (EDA) (≥99%), hexamethylendiamine (HMDA) (≥99%), propylamine (PA) (≥99%) and dibuthylamine (DBA) (≥99%) were supplied by Sigma-Aldrich (Munich, Germany).

### 2.2. Methodology

The experimental procedure for the non-isocyanate polyurethane (NIPU) synthesis consisted of mixing carbonated soybean oil (CSBO) together with different types of amines (PA, DBA, Priamines) in a 150 mL steel recipient after mild preheating of the reagents (50 °C), modifying the ratios between them both, and obtaining 40 g of NIPU product. For the case of ethanol/water modification, the preheating was raised to 70 °C. This mixture was stirred with a Heidolph RZH 2102 mechanical stirrer (2000 rpm) until its viscosity began to increase significantly, impeding further mixing. Once this point was reached, the mixture was placed on stainless-steel rectangular frames-shaped molds (120 × 100 × 2 mm). Then, these were introduced in a heating oven at a desired temperature and time to proceed to the curing of the samples.

### 2.3. Characterization Techniques

- Nuclear Magnetic Resonance (^1^H-NMR) Spectrometry

^1^H NMR spectra were recorded on a Bruker Ascend TM-500 spectrometer and referenced to the residual deuterated solvent using CDCl_3_ as a solvent. NMR spectra were acquired at 25 °C. Chemical shifts w given in ppm relative to CDCl3 (^1^H, 7.2 ppm).

- Infrared Spectrometry (FT-IR)

Infrared spectra were obtained with a Spectrum Two spectrometer (Perkin Elmer España S.L., Tres Cantos, Spain) with a universal attenuated total reflectance (UATR) accessory. The samples were scanned from 4000 to 450 cm^−1^ at a resolution of 16 cm^−1^. All measurements were performed at room temperature.

- Swelling Index (S. I.)

NIPU samples are cut out and immersed in tetrahydrofuran (THF) as a solvent for 24 h at room temperature. After this time, excess THF was removed, and the samples were weighted. The swelling index (S.I.) was calculated using Equation (1).

(1) $$\mathrm{Swelling}\;\mathrm{Index}\;\left(\%\right)=\frac{W_{s}-W_{0}}{W_{0}}\cdot 100$$

with W_0_ and W_s_ being the initial mass of the sample (mg) and its mass after solvent absorption (mg), respectively.

The swelling index is inversely related to the crosslinking degree of a polymer [42].

- Differential Scanning Calorimetry (DSC)

Thermal behavior of the NIPU samples was studied on a DSC 214 Polyma calorimeter (Netzsch) equipped with a refrigerated cooling system and nitrogen as the purge gas. Samples were analyzed with the following conditions:

– Nitrogen flow rate: 40 mL/min;
– Heating temperature range: 50–300 °C;
– Heating rate: 3 °C/min.

The data obtained were analyzed using TA Universal Analysis 2000 software.

- Thermogravimetric Analysis (TGA)

Thermal stability was studied on a STA 449 F3 Jupiter thermogravimetric analyzer (Netzsch) coupled to a QMS 403 Aëolos Quadro mass spectrometer (Netzsch). This system allows real weight changes to be obtained. Samples were analyzed with the following conditions:

– Nitrogen flow rate: 50 mL/min;
– Heating temperature range: 70–700 °C;
– Heating rate: 5 K/min.

The data obtained were analyzed using Proteus Analysis software.

- Tensile Strength

To determine the mechanical tensile strength of the synthesized elastomers, an MTS Criterion Model 43 was used. This equipment performs unidirectional tensile tests, the load crosshead speed used was according to the ASTM 412-D standard.

- Yield Calculations

CSBO carbonation percentage was calculated from epoxide and cyclic carbonate regions of ^1^H-NMR spectra. Relating their integral area to their relative amount, the reaction yield can be easily extracted from the spectra and calculated as a ratio between them, as explained in Equation (2).

(2) $$\mathrm{Yield}\;\left(\%\right)=\frac{A_{C}}{A_{E}\;+\;A_{C}}\cdot 100$$

A_C_: Integrated NMR signal value of cyclic carbonates (4.4–5.0 ppm).

A_E_: Integrated NMR signal value of epoxides (2.8–3.5 ppm).

## 3. Results and Discussion

The experimental work to obtain tailor-made NIPUs involved several stages. First, a preliminary study to set the standard operating conditions to carry out the NIPU synthesis was established.

Second, considering those standard conditions to synthesize the NIPU products, different tailor-made materials were synthesized using precursor diamines with clear structural differences, as the length of the hydrocarbon chain or the chemical structure. In addition, a chemical modification of the CSBO functionality, the cyclic carbonate source, is proposed, which allowed the range of products and properties obtained to be extended.

Finally, this synthesis pathway was adapted to a scenario in which all the raw materials used originate exclusively from bio-based resources.

### 3.1. Preliminary Experiments

A first group of experiments was carried out to establish the synthesis and curing conditions. The starting point of the planning is to study the reagents molar ratio (diamines/CSBO), which could be considered equivalent to the isocyanate index [6,43], but for NIPUs. For that, it is necessary to take into account the functionality of each one, that is, refer to the proportion between their functional groups.

For this purpose, a fully bio-based bifunctional amine (f = 2), derived from fatty acids (Priamine-1075), and carbonated soybean oil (CSBO), which presents several carbonyl functional groups, key for the achievement of the polymerization reaction, were used.

As the latter is a homogeneous mixture of compounds from mainly triglyceride origin, an average value for its functionality should be established (obtained from the original fatty acid profile of the oil, whose value is 4.37).

Thus, this proportion is calculated following Equation (3).

(3) ACC=molAmolCSBO·fAfCSBO

where A/CC is the molar ratio between reactants, mol_A_ is the molar amount of amine (Pr-1075), mol_CSBO_ is the molar amount of the cyclic carbonate source (CSBO) and f is the functionality of each of them.

The synthesis conditions were then first optimized with a range of samples subjected to different curing times (0–24 h) and a fixed temperature (100 °C), based on the methodology proposed by Błazek et al. (2020) for similar purposes [42].

#### 3.1.1. Influence of the Amine/Cyclic Carbonates Molar Ratio

**Figure 2:**
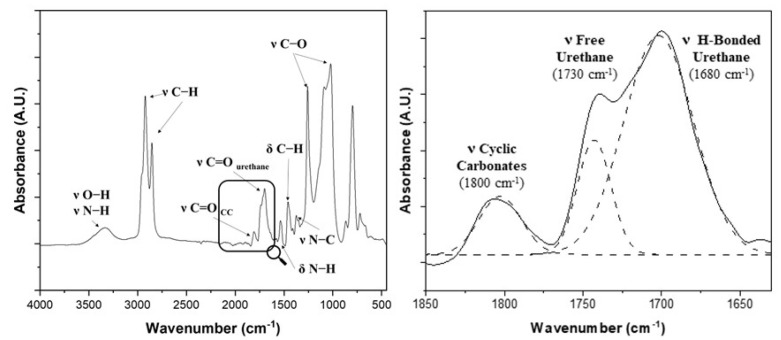
FT-IR NIPU spectra and carbonyl urethane/CC deconvolution (T = 100 °C, A/CC = 1, t = 30 min).

FTIR characteristic spectra of the NIPU samples with the detailed deconvolution of cyclic carbonate and urethane carbonyl (C=O) are shown in Figure 2.

Apart from those two signals (C=O carbonyls, 1600–1830 cm^−1^), characteristic NIPU FT-IR signals were also identified: O-H and N-H stretching bands at 3170–3550 cm^−1^, C-H stretching vibrations (2800–3000 cm^−1^), N-H deformations (1515–1535 cm^−1^), C-H bending vibrations (1420–1450 cm^−1^), C-N stretching vibrations (1350–1390 cm^−1^) and C-O (1000–1200 cm^−1^), related to urethane linkages [38,42].

**Figure 3:**
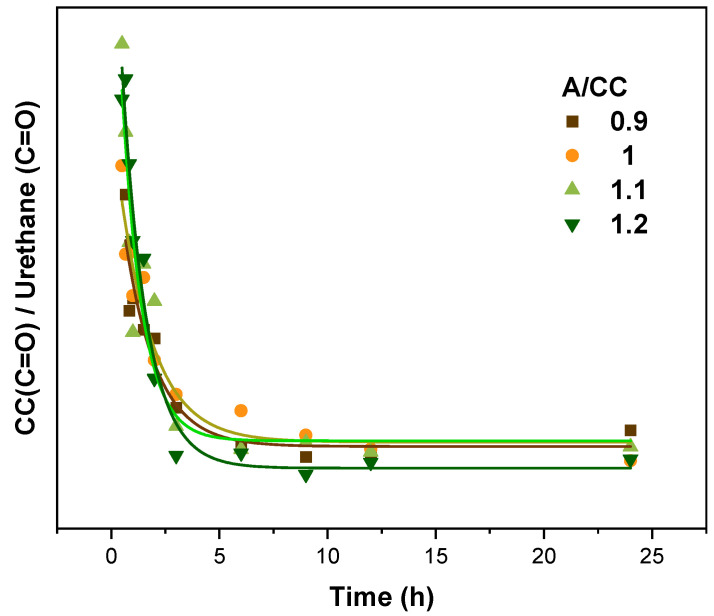
FT-IR integrated data for the development of the NIPU curing process. (T = 100 °C, A/CC = 1, t = 0–24 h).

From the infrared spectrum, it is possible to qualitatively follow the development of the curing process, and for this purpose, the mentioned deconvolution method using a Gaussian model and subsequent integration of the area of the peaks corresponding to the carbonyl associated with urethane groups (1600–1750 cm^−1^) and the cyclic carbonates (CC) (1770–1830 cm^−1^), is applied. The evolution of the CC/urethane area ratio is shown in Figure 3.

As can be seen in Figure 3, the curing process advances exponentially until a moment at which the area representing the cyclic carbonates can be considered residual and the process can be considered complete. This fact can also be appreciated through visual/touch observation by the firmness and surface stiffness of the samples.

**Figure 4:**
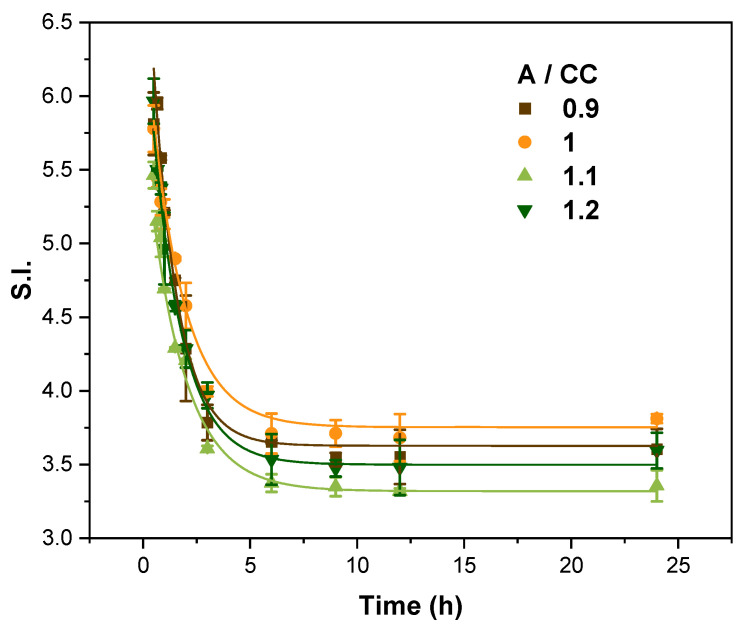
A/CC molar ratio swelling index results within a 24 h curing time (T = 100 °C).

In order to obtain quantitative data to draw conclusions and establish the optimal conditions for the synthesis of NIPUs, swelling index measurements were performed on all samples. Figure 4 compares the results of these measurements.

As mentioned in the experimental section, swelling behavior is inversely related with the crosslinking degree of a polymer [44], so as the crosslinking advances with the course of the polymerization a lower S.I. is observed. The observations of crosslinking also confirm that the polymerization is essentially finished in 6 h.

Considering these results, the optimal molar ratio value is the one that reaches a lower final value of swelling index, with total cure of the material and a crosslinking level that let a wider range for further modification. Therefore, it is decided to set the A/CC molar ratio at a value of 1.1 for subsequent experiments.

#### 3.1.2. Curing Temperature Influence

**Figure 5:**
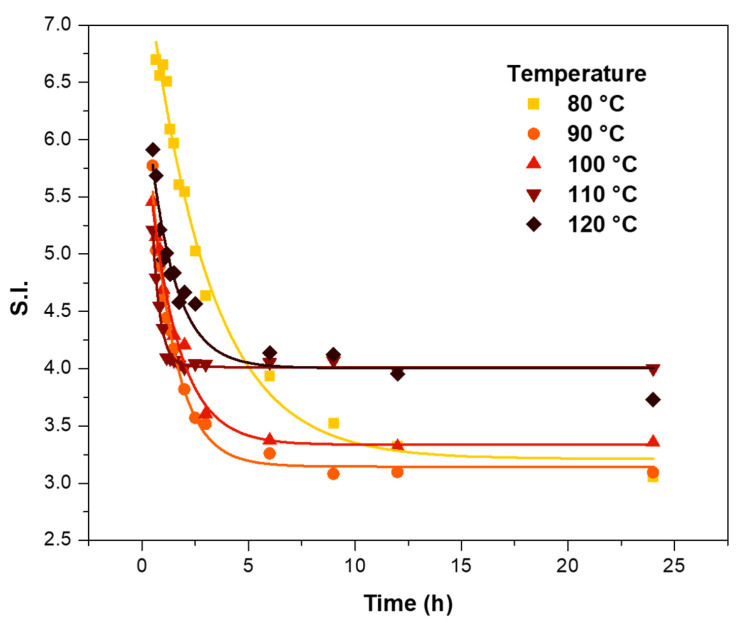
Temperature influence on swelling index results (A/CC = 1.1, t = 0–24 h).

The effect of temperature on the curing time was studied in the range of 80–120 °C (Figure 5).

In Figure 5, it can be clearly seen that it is not efficient to raise the temperature to reduce curing times. Hence, 90 °C can be a good choice because an earlier complete curing time (6 h) and appropriate final crosslinking degree are achieved.

In conclusion, the best combination of synthesis and curing conditions could be 6 h of reaction time, 90 °C and an A/CC ratio of 1.1.

### 3.2. Tailor-Made Non-Isocyanate Polyurethane (Monoamine Blocking Method)

Once the specific methodology to obtain NIPUs from CSBO and diamines was established, the objective of the research focused on widening the range of product properties that can be obtained from the same raw material. For this purpose, modification of the structure of these reagents is proposed, in order to obtain products with different properties that can be tuned at will.

**Scheme 2:**
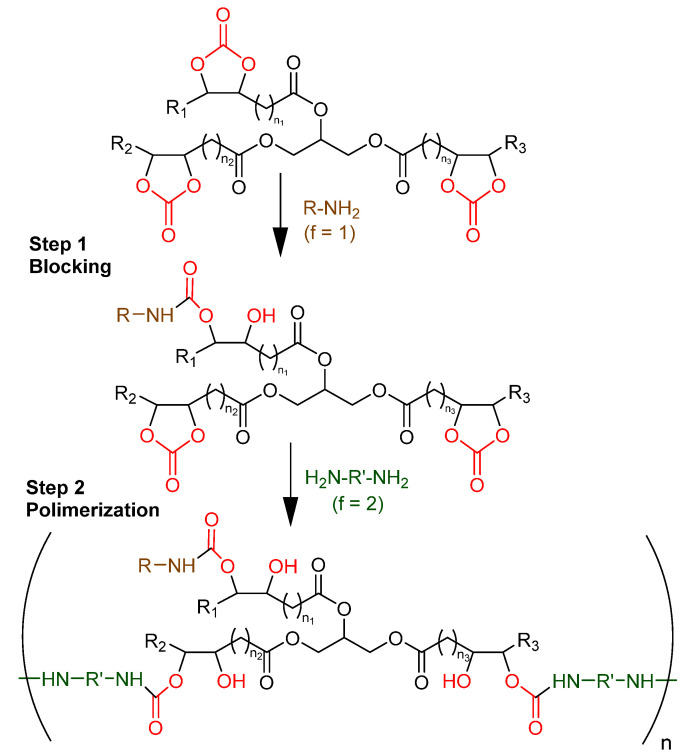
Multistep modified-functionality NIPU synthesis mechanism (blocking method). Cyclic carbonate groups and derivatives are in red. Amine groups in green.

The proposal consists of the “blocking” part of the functional groups of the CSBO. This blocking is achieved by contacting CSBO with monofunctional amines (f = 1), leaving growing chains and crosslinking available only for those that have not reacted. The proposed process is explained in Scheme 2.

Scheme 2 describes a multi-step mechanism in which initially the monofunctional amine is added, which will be responsible for modifying the functionality of the cyclic carbonate source. This first step is then adjusted stoichiometrically, following Equation (4):

(4) $${f^{\prime }}_{\mathrm{CC}\;}{=f}_{\mathrm{CC}}\cdot \left(1-\frac{{\mathrm{mol}}_{\mathrm{CC}}}{{\mathrm{mol}}_{A^{1}}}\right)$$

with f′_CC_ being the targeted functionality of CSBO after modification, f_CC_ the initial functionality of CSBO (4.37), mol_CC_ the contributed moles of carbonyl groups and mol_A_^1^ the contributed moles of monofunctional amine.

After this modification, the polymerization is completed through the addition of the corresponding amount of diamine (Equation (5)).

(5) $${\mathrm{mol}}_{A^{2}}{=f^{\prime }}_{\mathrm{CC}}.{\mathrm{mol}}_{\mathrm{CC}}$$

with mol_A_^2^ being the contributed moles of the difunctional amine.

For this purpose, NIPUs are synthesized from the following:

- Carbonated soybean oil (CSBO).
- Propylamine (PA) and dibutylamine (DBA) as monofunctional amines. Both primary and secondary amines are selected in order to study their influence on the blocking step.
- Ethylenediamine (EDA), hexamethylenediamine (HMDA), and Priamine™ 1075 (Pr-1075) as difunctional amines acting as chain extenders. The difference between each of them lies in their molecular weight and thus in the length of the chains, allowing the study of their effect on the final properties of the material.

Several NIPUs are formulated (f′_CC_ = 2, 2.5, 3, 3.5 and 4) by reacting CSBO with every difunctional amine (A/CC = 1.1) and using both blocking agents (monoamines). Curing was carried out at 90 °C for 6 h.

The NIPU corresponding to EDA (f′_CC_ = 2) showed severe curing and solidifying issues, which avoided its completion. The low functionality intended for that system could have caused some of the oil molecules to have one or even zero functionalities remaining as discrete oil molecules or oligomers.

**Figure 6:**
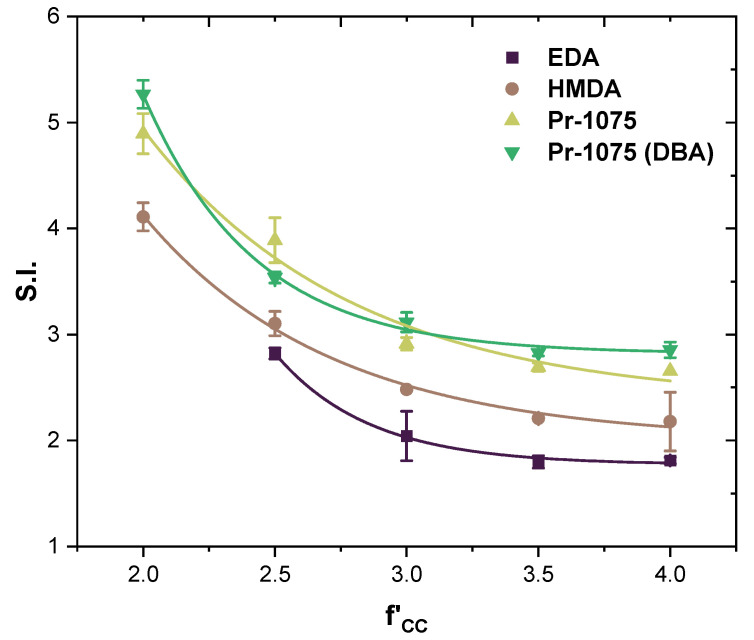
Swelling index results for the f′_CC_ study.

The results of the characterization of the swelling index for the different types of amines are shown in Figure 6.

It can be seen that those systems with a lower functionality present a greater swelling related with a lower crosslinking approaching zero for f′cc = 2 (nearly a thermoplastic material). Additionally, the greater swelling and chain mobility can be related not only to a decrease in the network crosslinking, but also to the reduction of the physical intermolecular interaction between chains, mainly hydrogen bonds (between -NH and -C=O) [39], which can be attributed, in part, to the greater distance between the urethane groups from different chains.

More noteworthy aspects are that the differences in reactivity between the NIPUs synthesized using secondary amines (DBA) and primary amines (PA) are hardly appreciable. This is indicative of the fact that by simply slightly preheating (50 °C) the reagents and maintaining a sufficient stirring time for stage 1 (blocking), the reactivity of the secondary amine is ensured, which also allows complete conversion.

It is also observed how the molecular weight of the selected diamine has an influence on the swelling and chain mobility (stiffness). When shorter amine chain segments are employed, the H-bond number is greater because the distances between H-bond promotors (like urethanes groups) are also reduced, and therefore the polymer swelling decreases [45].

**Figure 7:**
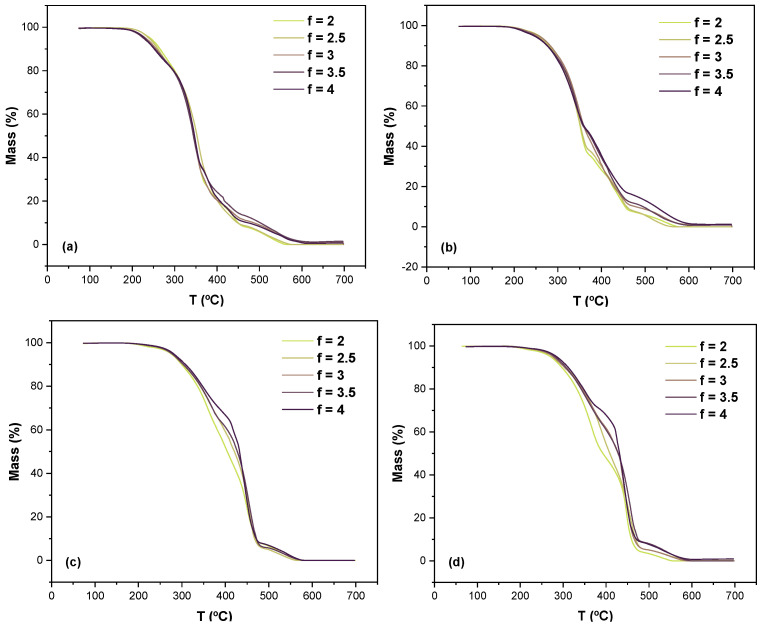
TGA analysis results. (**a**) EDA, (**b**) HMDA, (**c**) Pr–1075, (**d**) Pr–1075–DBA.

Subsequently, the thermal behavior of the NIPUs was evaluated. Figure 7 shows the TGA plots of the synthesized samples.

Three different stages of mass loss are observed: the first one (270–350 °C) associated with the breakage of urethane, the second one (350–450 °C) with the degradation of ester and amide bonds and, finally, the last one (450–570 °C) assigned to aliphatic chain decomposition.

As the crosslinking density increases, both the physical and chemical resistance increases, as a result of a more strongly interconnected three-dimensional network that requires more energy to degrade. Similarly, the lower the molecular weight of the amine and the smaller the size of the monomer segments, the lower the structural mobility, i.e., it is much more constrained and stronger bonds with high energetic stability are created, being almost negligible in the case of EDA.

TGAs also confirms the good stability of the NIPUs, since the degradation of the material is not appreciable below 250 °C.

**Figure 8:**
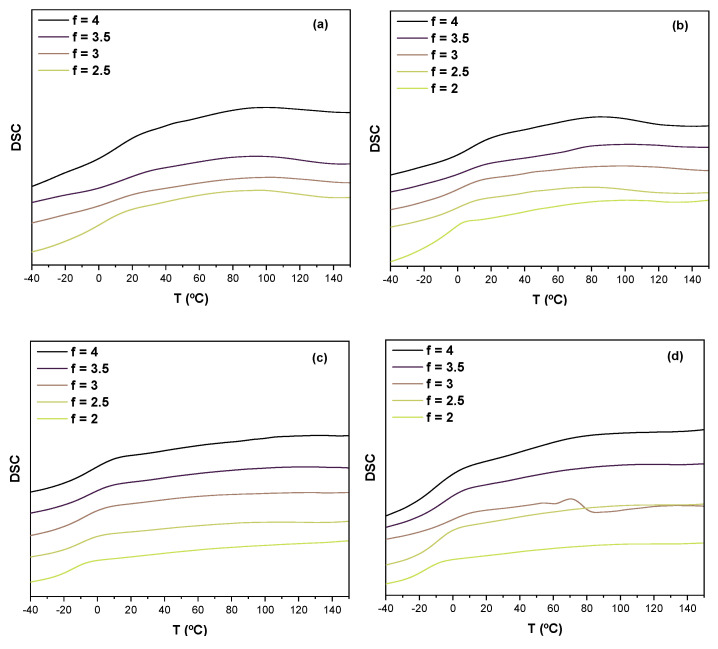
DSC analysis results. (**a**) EDA, (**b**) HMDA, (**c**) Pr–1075, (**d**) Pr–1075–DBA.

To complete the thermal characterization, DSC analysis of the samples was performed (Figure 8).

**Figure 9:**
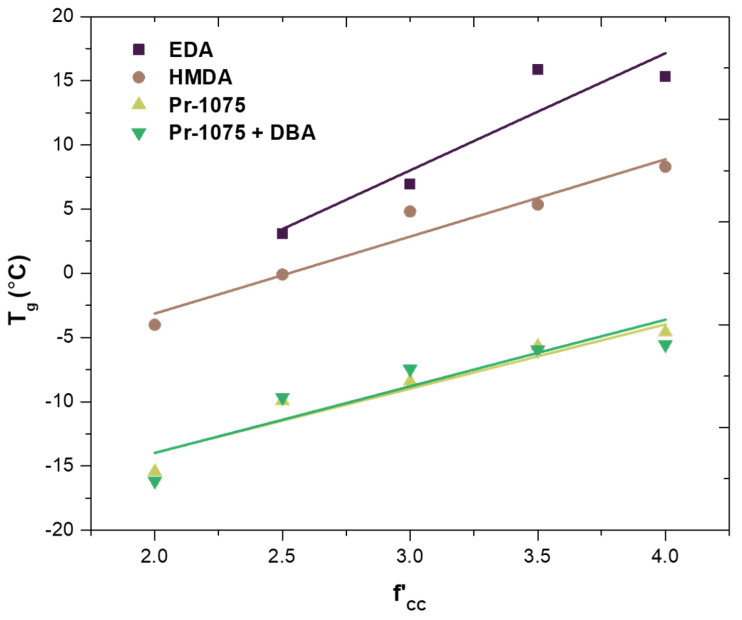
Glass transition temperature (Tg) NIPU values.

The glass transition temperature is the most relevant data can be extracted from the analysis and the values obtained are represented in Figure 9.

The T_g_ obtained values are lower than those of conventional polyurethanes, but similar to those of vegetable oil-derived NIPUs, indicating that they are mostly amorphous. This might be attributed to the long branched fatty acid chains, which may have inhibited the crystallization of the polymers [46].

However, T_g_ decreases with the molar mass of the precursors, and increases with the crosslinking density of the NIPUs [19].

**Figure 10:**
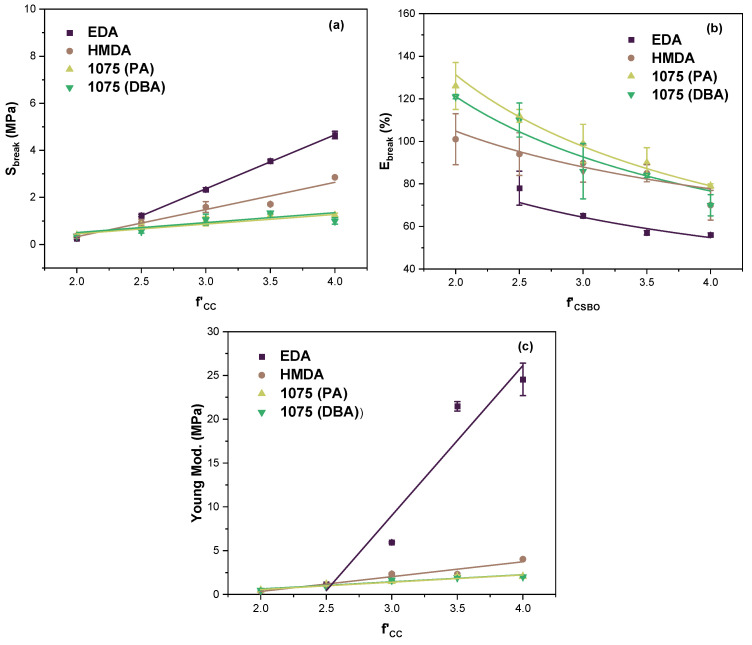
Tensile strength results (ASTM 412-D). (**a**) Stress at break, (**b**) elongation at break, (**c**) Young’s modulus.

Finally, another important factor that defines further applications is the tensile strength (elasticity/stiffness), measured according to ASTM D-412 (Figure 10).

As expected, Figure 10 shows that elasticity (which is also inversely related to tensile strength) is greater for low crosslinking values with a large freedom of movement [44], which is linked to the linearity of the polymers that allows for a better flow of the polymer and results in materials with a higher elongation capacity [47]. In addition, the used diamine molecular weight has a clear influence, resulting in a higher stiffness as the molecular weight of the diamine decreases, especially in the case of ethylenediamine. This behavior could be understood analogously to the effect produced by the drop in molecular weight of the polyol that usually acts as a soft segment in traditional polyurethane elastomers strengthening the hydrogen bonding [38].

### 3.3. Fully Bio-Based Tailor-Made Hybrid Non-Isocyanate Polyurethane (Hydroxyl Blocking Method)

The last stage of the research on the production of tailor-made NIPUs consists of adapting the chemical modification process to one that only uses raw materials of strictly renewable origin.

To achieve this goal, the use of partially carbonated or hybrid soybean oil (H-CSBO) and water or ethanol as blocking agents is proposed.

Hybrid oils are characterized by the presence of different functional groups as active sites, such as epoxides and cyclic carbonates. To obtain these oils, a methodology developed in previous publications was followed, in which a specific percentage of the starting epoxide groups present in the epoxidized soybean oil (ESBO) can be converted simply by adjusting the operating conditions. Those partially carbonated epoxidized oils are commonly used to obtain what is known as hybrid NIPUs or HNIPUs [48].

**Scheme 3:**
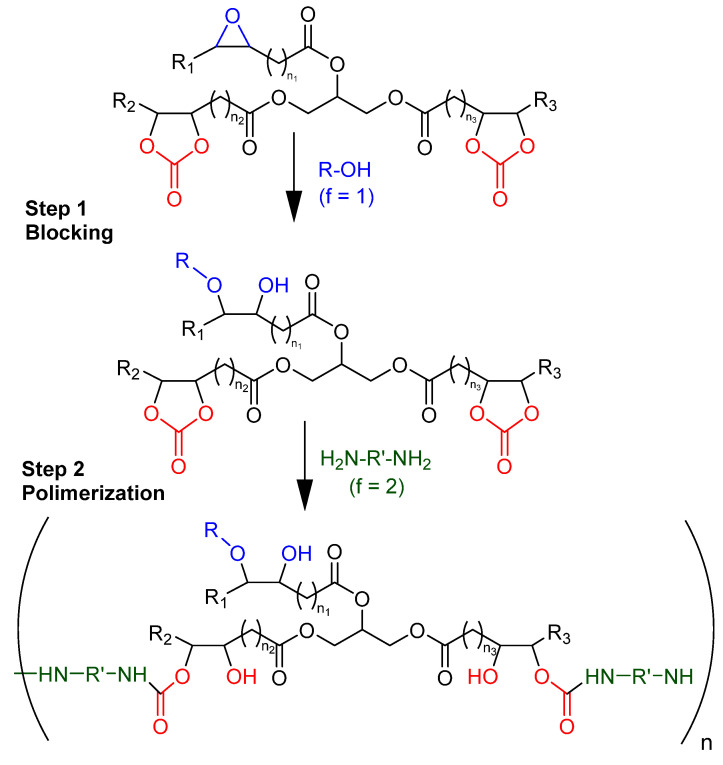
Hybrid NIPU synthesis (f′_CC_ tunning). Cyclic carbonate groups and derivatives are in red. Amine groups in green. Epoxide groups and derivatives in blue.

However, in this research, to use this raw material with another approach is intended, consisting of making it react in the first instance, to directly block the remaining oxirane rings, with nucleophilic compounds that have terminal hydroxyls, such as ethanol or water, thus eliminating the need to use compounds derived from non-renewable sources, such as propylamine, in the process of controlling the crosslinking of these polymers. The proposed reaction mechanism is shown in Scheme 3.

Therefore, in order to test the feasibility of this method, the synthesis of different bio-based polyurethane materials using partially carbonated oil as a raw material is proposed, with a conversion value that allows the study of a wide range of functionalities, as shown in Table 1 Priamine^TM^ 1075, as diamine polymerization agent, and ethanol or H_2_O, as blocking agents, were selected to obtain a fully bio-based hybrid non-isocyanate polyurethane.

**Figure 11:**
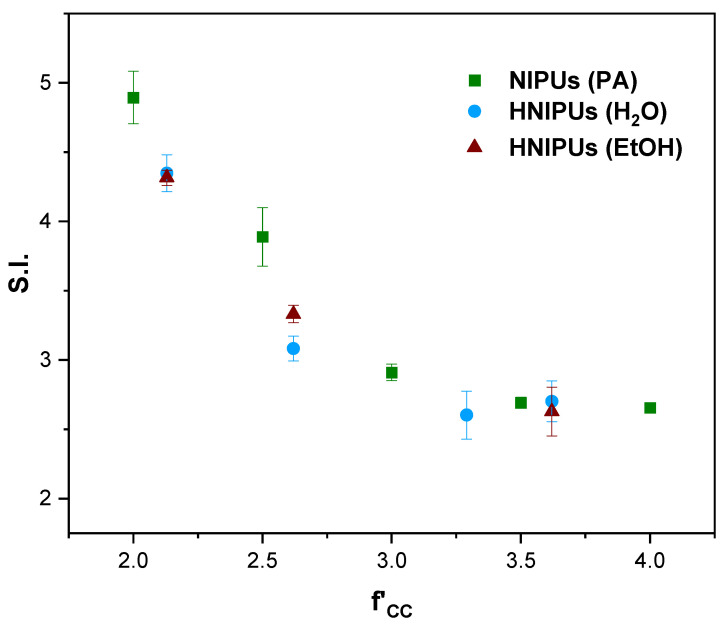
Swelling index comparative results of NIPU–HNIPUs.

**Figure 12:**
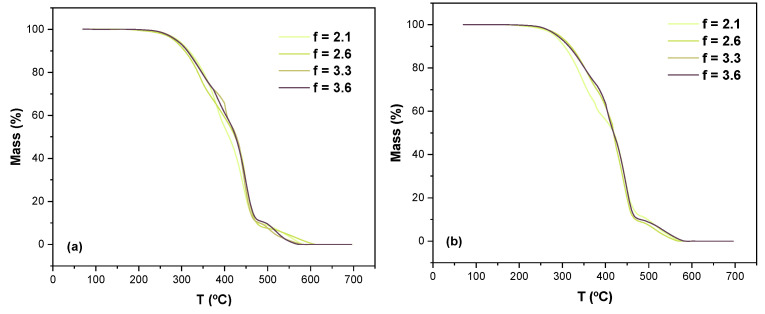
HNIPUs TGA analysis. (**a**) HNIPUs (H_2_O), (**b**) HNIPUs (EtOH).

**Figure 13:**
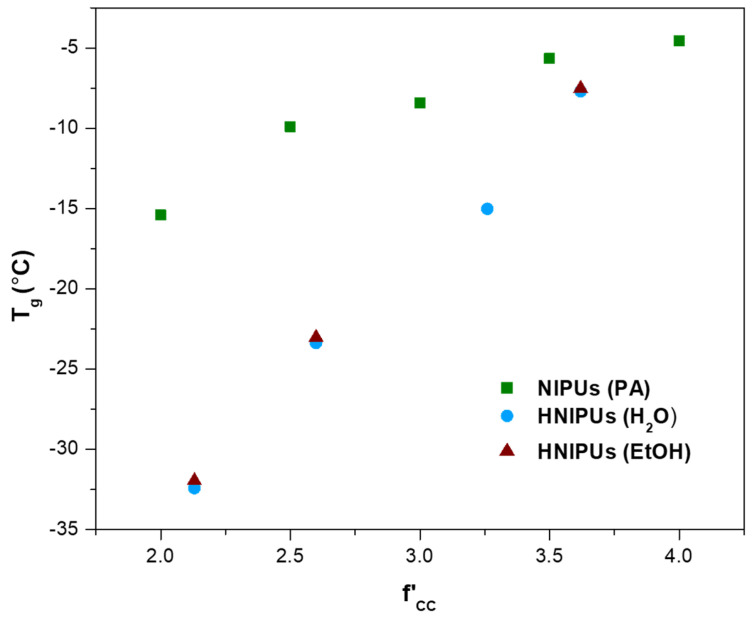
Glass transition temperature (T_g_) comparative values for NIPU-HNIPUs.

**Figure 14:**
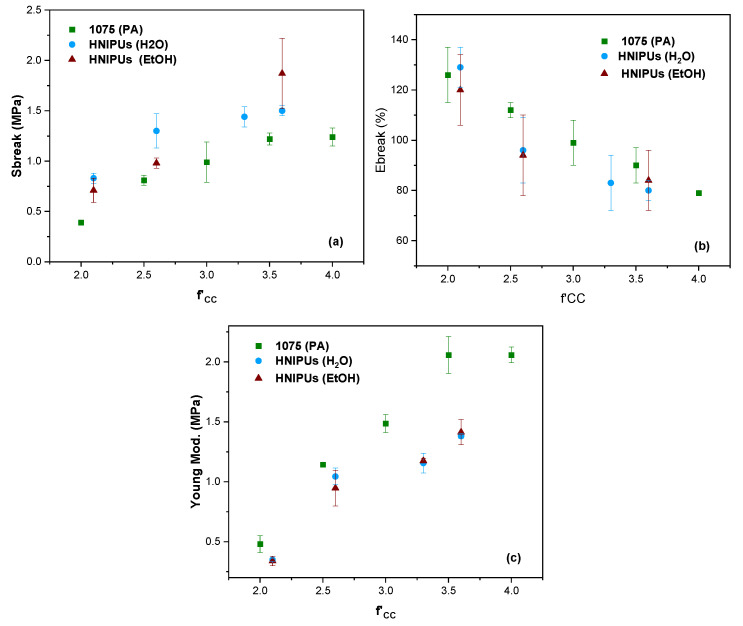
Tensile strength results for H-NIPUs (ASTM 412-D). (**a**) Stress at break, (**b**) elongation at break, (**c**) Young’s modulus.

The characterization procedure followed for the rest of the materials analyzed in this research article is the same, starting with the analysis of the crosslinking density (Figure 11), their thermal properties (Figure 12 and Figure 13) and, finally, their mechanical properties (Figure 14).

The decrease in the swelling index with functionality, as can be seen in Figure 11, confirms that the method for the adjustment of functionality of fully carbonated oils also works for hybrid oils.

**Figure 15:**
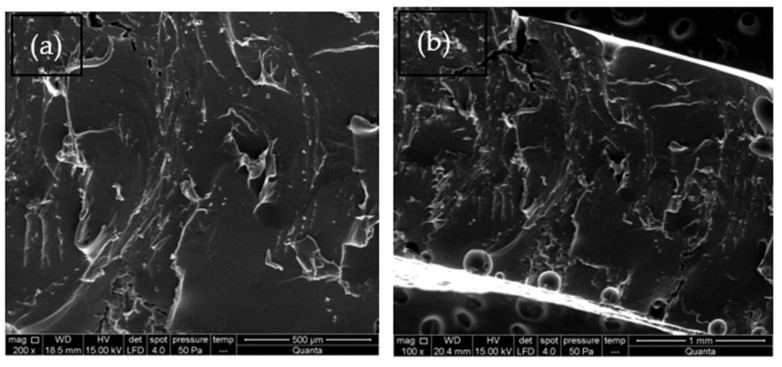
SEM photographs of the internal structure of the NIPUs. (**a**) Inner part, (**b**) full section.

The curing temperatures used for H_2_O and ethanol as blocking agents were set at 90 °C (never surpassing this temperature), and enough time was given for the release of possible bubbles, as this is the first step in the two-step synthesis process. In Figure 15, it can be seen, as an example, that no bubbles or trapped gas were observed in the core of the NIPU film obtained; there are only a few small, superficial bubbles that should not have any significant implication on the structural, mechanical or thermal properties of the material. The absence of gas inside the polymers was also proved by density measurements, showing a range of values from 920 to 946 kg m^−3^ for the prepared samples.

The thermal and mechanical behavior (Figure 12 and Figure 14) of these materials is also very similar to those obtained by the monoamine-blocker method for CSBO, with both the values of swelling and global trends being also comparable. The most significant differences are observed for the glass transition temperatures (T_g_) (Figure 13) where there is a relative decrease in the new materials with respect to those obtained with the previous method. This behavior is due to the hydroxyl blocking of the oxirane rings, which undergoes hydrolyzation of the epoxide, but no formation of urethane linkages is observed, as in the case of the monoamine blocking method.

For each characterization method, the values obtained for the HNIPUs synthesized with either water or ethanol (with the same f′_CC_) are essentially identical, thus allowing the distinct use of either of them. This exemplifies that this synthetic route is able to generate structure-modified NIPUs with the advantage of fully renewable components, such as the amines used as blocking agents, replacing them with an extremely simple, cheap and 100% eco-friendly reagent, such as water itself, thus also allowing the materials obtained to be of completely renewable origin.

Table 2 summarizes the biobased content of the NIPUS synthesized in this work in relation to their composition, finally achieving a 100% bio-based polyurethane.

## 4. Conclusions

Isocyanate-free polyurethane elastomeric materials from renewable raw materials were successfully synthesized, using carbonated soybean oil (CSBO) and Priamine^TM^1075, a product derived from dimerized fatty acids.

Optimal conditions and a methodology for the synthesis and curing of the materials were then established, setting them at a molar A/CC ratio of 1.1 and a temperature and curing time of 90 °C and 6 h, respectively.

A modification method of the precursors’ chemical structure was developed, by means of a procedure that has not been previously published, that proposes the partial blocking of the polymerization active sites, which allows materials to be obtained with a wide range of properties that are adjustable according to specific needs.

Hybrid NIPUs od 100% renewable origin can be synthesized, eliminating the use of amines derived from non-renewable sources.

## Figures and Tables

**Table 1:** Hybrid CSBO conversion and functionality values.

Sample	CC Conversion (^1^H-NMR)	f’_CC_
1	48.9	2.1
2	60.0	2.6
3	74.9	3.3
4	82.2	3.6

Curing: t = 6 h, T = 90 °C, A/CC = 1.1.

**Table 2:** Bio-based NIPU raw material content (%wt).

Bio-Based Origin (%wt)				
f′_CC_	1075 (PA)	1075 (DBA)	HMDA	EDA
4.37	100.00%	100.00%	81.00%	89.18%
4	99.01%	97.87%	80.97%	88.39%
3.5	97.56%	94.81%	80.94%	87.36%
3	95.95%	91.54%	80.91%	86.34%
2.5	94.15%	88.03%	80.88%	85.35%
2	92.12%	84.37%	80.85%	84.39%
H-NIPUs	100.00%			

## Data Availability

The data presented in this study are available on request from the corresponding author. The data are not publicly available due to privacy.
